# A novel pathogenic *CACNA1A* variant causing episodic ataxia type 2 (EA2) spectrum phenotype in four family members and a novel combined therapy

**DOI:** 10.1007/s00415-020-10190-1

**Published:** 2020-09-10

**Authors:** Josef Penkava, S. Ledderose, S. Chahrokh-Zadeh, A. Munzig, zu Eulenburg, D. Huppert, M. Strupp, S. Becker-Bense

**Affiliations:** 1 German Center for Vertigo and Balance Disorders (DSGZ), Ludwig-Maximilians-Universität München, Campus Großhadern, Marchioninistr. 15, 81377 Munich, Germany; 2 Department of Pathology, Ludwig-Maximilians-Universität München, Munich, Germany; 3 Center for Human Genetics and Laboratory Diagnostics (CHGLD), Martinsried, Germany; 4 Department of Neurology, Ludwig-Maximilians-Universität München, Munich, Germany

Dear Sirs,

Episodic ataxias (EAs) are rare neurological disorders characterized by recurrent episodes of cerebellar ataxia with an imbalance of stance and gait, limb ataxia, dysarthria, and nystagmus, often triggered by physical or emotional stress, or alcohol and accompanied by nausea and vomiting (for review see [1, 2]). Currently, there are at least seven known subtypes of EA. Among them, EA1 and EA2 are clinically most relevant [2]. EA2 has its onset typically in adolescence, but some cases with a late onset have been reported [3]. EA2 episodes generally last between minutes and hours, and are accompanied by migraine-like cephalgia in around 50% of patients [4]. EA 2 patients frequently develop slowly progressive interictal ataxia and distinct central ocular motor dysfunctions, e.g., mainly gaze-evoked or downbeat nystagmus [4, 5]. EA2 is an inherited autosomal-dominant channelopathy, caused by pathogenic variants affecting the *CACNA1A* gene on chromosome 19p13, which encodes the alpha-1A subunit of the P/Q-type voltage-gated calcium channel (Cav2.1) [1, 6]. The latter is found ubiquitously in the nervous system with high expression levels in cerebellar Purkinje cells [7, 8]. EA2 shares molecular pathologic similarities with spinocerebellar ataxia type 6 (SCA6), familial hemiplegic migraine (FHM) and epileptic encephalopathy, also carrying mutations in the *CACNA1A* gene. Typically, cases of FHM present with missense mutations, SCA6 with a C-terminal polyglutamine expansion and EA2 harbors point mutations resulting in premature stop codons as well as small or large deletions, insertions and missense mutations [2]. During the last decades, an increasing number of *CACNA1A* variants have been described. Here, we report on a female patient with a novel pathogenic *CACNA1A* variant and give insights into the heterogeneous phenotypes within her family.

A 47-year-old white Caucasian female presented to our tertiary outpatient center with recurrent attacks of postural imbalance (no vertigo sensu stricto*,* no nausea or vomiting) with the feeling of falling forward, and an associated holocephalic headache that began around the age of 15 years. The attacks occurred daily, most often in stressful situations and usually lasted for several hours. No other triggers were evident. By the time the patient presented to us, she felt permanently dizzy and posturally unstable in between the attacks, rendering her dependent on regular help in her daily routine. There was no catamnestic report of seizures or syncopes.

Her clinical and neuro-ophthalmological examination revealed a cerebellar ocular motor dysfunction with bilateral horizontal gaze-evoked and rebound-nystagmus, saccadic pursuit in all directions, hypermetric horizontal and vertical saccades, reduced optokinetic nystagmus in all directions, and an most probably centrally impaired horizontal vestibulo-ocular reflex bilaterally (video head-impulse test: gain of 0.44 on the right and 0.35 on the left). Bithermal caloric testing showed a central preponderance of left-beating nystagmus without spontaneous nystagmus (warm right – 3.8°/s, warm left + 22.9°/s, cold right 6.4°/s, cold left – 2.0°/s). Finger-to-finger following showed slightly hypermetric movements. Romberg’s test revealed an imbalance in tandem stance. Quantitative gait analysis with the GAITRite system objectified a dynamic instability due to incipient impaired cerebellar postural adjustment. Cranial MRI showed several small supratentorial white matter lesions, but no distinct vermian atrophy as is sometimes reported in EA 2 [1].

Genetic testing using next-generation sequencing and Sanger sequencing methods (i.e. enrichment of the genes *CACNA1A, CACNB4, KCNA1, SCN2A, *and *SLC1A3*, followed by next-generation sequencing of coding exons as well as conserved parts of splice sites, and bioinformatic data analysis; hereby, in a first step an analysis of variants of the *CACNA1A* gene was performed prior to confirmation of the variant by amplification with polymerase chain reaction and analysis by Sanger sequencing after a second, independent DNA extraction from the original blood sample) revealed a novel, heterozygous, pathogenic [9, 10] variant in exon 16 of the *CACNA1A* gene NM_001127221.1: [c2070_2071delinsGGAG, p.(Phe690Leufs*9)]. Deletions of cytosine and thymidine at position 2070–2071 and insertion of four nucleotides at this position in exon 16 of the *CACNA1A* gene were identified. This leads to a frameshift during translation and to an early stop of protein synthesis at codon position 698 after the incorporation of eight changed amino acids. This variant has so far not been described in either the literature or has been observed in genome sequencing data from large-scale sequencing projects, e.g., ExAC (Exome Aggregation Consortium) or GnomAD (Genome Aggregation database). Other translational stop variants in the *CACNA1A* gene have been described in EA2 patients before [11, 12].

The same heterozygous *CACNA1A* variant was detected in three family members (Fig. 1) of our index patient, but the clinical phenotypes varied considerably. The 68-year-old mother is suffering from a slowly progressive imbalance of gait for almost 20 years. She negated cephalgia, episodic ataxia or vertigo. Her imbalance of gait is not accompanied by vegetative symptoms such as nausea or emesis. There are no triggers evident. Neurological examination of the oculomotor system revealed a gaze-evoked nystagmus to the right side. Romberg’s test showed omnidirectional swaying and finger-to-nose testing showed a slight dysmetria on both sides. Her gait was broad based and atactic. She has been taking acetazolamide (250 mg/days) for about 3 months with no significant amelioration of her symptoms. The 45-year-old brother of our patient reported intermittent cephalgia, which started in his early childhood. He negated any recurrent attacks of postural imbalance, ataxia or vertigo. He does not take any medication regularly apart from NSAR temporarily. Neurological examination was without pathological findings. The 15-year-old nephew of our patient (her brother’s son) has suffered from episodic attacks with imbalance of gait, vertigo, and headache, typically lasting for several hours and triggered by stress since the age of 14 years. In between the attacks, he feels asymptomatic.

**Fig. 1:**
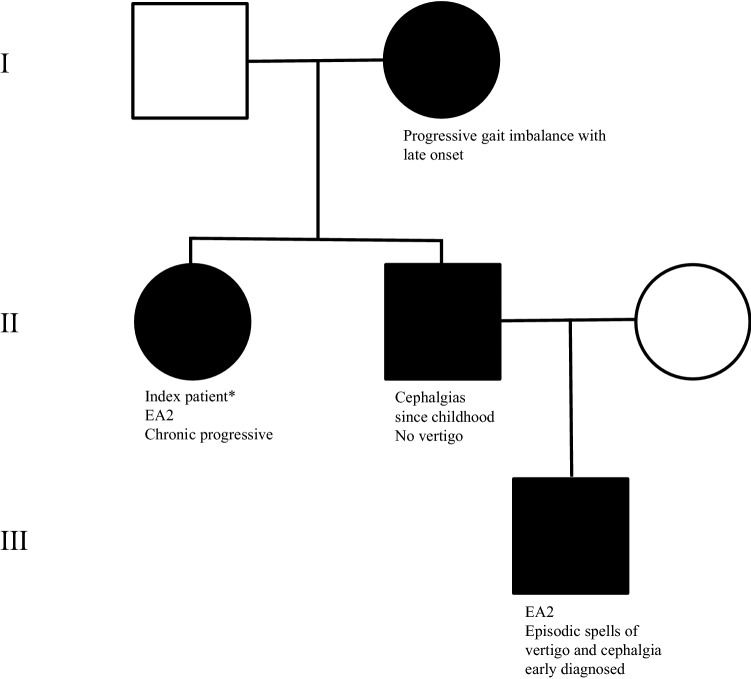
Pedigree of a German family carrying the novel pathogenic heterozygous autosomal dominant *CACNA1A* variant. The newly identified heterozygous variant NM_001127221.1: [c2070_2071delinsGGAG, p.(Phe690Leufs*9)], was found in four family members in three generations showing a heterogeneous phenotype. Legend: “Asterisk” = index patient, square = male, circle = female, filled symbol = genetically affected subject, blank symbol = genetically unaffected subject

Pathogenic variants in the *CACNA1A* gene are known to lead to a broad clinical spectrum including EA2, SCA6, FHM and epileptic encephalopathy. Different variants usually tend to associate with different phenotypes, but sometimes family members harboring the same variant show a wide variability of the neurological manifestations [4, 13]. One further reason for the heterogeneous phenotype in our reported family could be incomplete penetrance. There is substantial evidence of intra-familial incomplete penetrance due to a pathogenic *CACNA1A* variant in the literature [4, 14]. Nevertheless, an involvement of other genes and epigenetic mechanisms cannot be excluded.

Therapeutic principles in patients with EA include medical treatment and physiotherapy, occupational therapy to preserve gait function, and speech therapy [15, 16]. In a randomized placebo-controlled trial, it was shown that 4-aminopyridine [17] is effective for the symptomatic treatment of EA2 (and downbeat nystagmus) [18]. Our patient did not respond sufficiently to the medical monotherapeutic treatments of choice with 4-aminopyridine (Fampyra™ 20 mg/days) and acetazolamide (250 mg–500 mg/days, discharged due to intolerable side effects such as paresthesia and kidney dysfunction) [19]. The modified amino acid acetyl-dl-leucine has been introduced as a drug for the symptomatic treatment of cerebellar disorders [20, 21]. Under reduction of 4-aminopyridine, the patient indicated significant aggravation of her previously described symptoms. Consequently, we reestablished the well tolerated daily dose of 4-aminopyridine (20 mg/days). However, the effect of the medication was not fully satisfactory to the patient. Therefore, we applied *ex juvantibus* a combined individual therapeutic approach with 4-aminopyridine (Fampyra™ 20 mg/days) and acetyl-dl-leucine (5 g/days) in a second step. Over the ensuing observation period of 12 months, a subjectively relevant improvement of the patient’s daily performance, accompanied by a stabilization of objective clinical and functional measures such as videooculography (no deterioration of ocular motor function), gait analysis (increase in velocity and step length) and ataxia scores (SARA score 4) could be observed interictally.

In conclusion, we identified a novel, pathogenic heterozygous *CACNA1A* variant NM_001127221.1: [c2070_2071delinsGGAG, p.(Phe690Leufs*9)] in a patient with EA2 and three other family members with a long undiagnosed history of the illness and a very heterogeneous phenotype across three generations of the family carrying the identical variant. The analysis of novel *CACNA1A* variants is an essential step in the in-depth characterization of EA2 patients. Our results might serve for molecular-genetic testing in similar patients, albeit future genetic analyses will most likely use high-throughput sequencing. From a therapeutic perspective after the patient did not respond sufficiently to standard treatment, a novel combination of 4-aminopyridine (Fampyra™ 20 mg/days) to reduce the frequency of attacks, and acetyl-dl-leucine (5 mg/days) to symptomatically alleviate interictal cerebellar ataxia resulted in subjective improvement and no further progression of the disease to date. Such combined therapies might have an over-additive effect because of different modes of action and may open up a new treatment path in EA2 individuals, especially in cases where 4-aminopyridine or acetazolamide medication alone does not alter the course of the disease. However, further randomized placebo-controlled trials in episodic ataxias are needed.

## References

[1] Kipfer S, Strupp M (2014). The clinical spectrum of autosomal-dominant episodic ataxias. Mov Disord Clin Pract.

[2] Jen JC, Wan J (2018). Episodic ataxias. Handb Clin Neurol.

[3] Imbrici P, Eunson LH, Graves TD, Bhatia KP, Wadia NH, Kullmann DM, Hanna MG (2005). Late-onset episodic ataxia type 2 due to an in-frame insertion in *CACNA1A*. Neurology.

[4] Jen J, Kim GW, Baloh RW (2004). Clinical spectrum of episodic ataxia type 2. Neurology.

[5] Riant F, Vahedi K, Tournier-Lasserve E (2011). Hereditary episodic ataxia. Rev Neurol (Paris).

[6] Ophoff RA, Terwindt GM, Vergouwe MN, van Eijk R, Oefner PJ, Hoffman SM, Lamerdin JE, Mohrenweiser HW, Bulman DE, Ferrari M, Haan J, Lindhout D, van Ommen GJ, Hofker MH, Ferrari MD, Frants RR (1996). Familial hemiplegic migraine and episodic ataxia type-2 are caused by mutations in the Ca2+ channel gene *CACNL1A4*. Cell.

[7] Jen JC, Graves TD, Hess EJ, Hanna MG, Griggs RC, Baloh RW (2007). Primary episodic ataxias: diagnosis, pathogenesis and treatment. Brain.

[8] Isaacs DA, Bradshaw MJ, Brown K, Hedera P (2017). Case report of novel *CACNA1A* gene mutation causing episodic ataxia type 2. SAGE Open Med Case Rep.

[9] Richards S, Aziz N, Bale S, Bick D, Das S, Gastier-Foster J, Grody WW, Hegde M, Lyon E, Spector E, Voelkerding K, Rehm HL (2015). Standards and guidelines for the interpretation of sequence variants: a joint consensus recommendation of the American College of Medical Genetics and Genomics and the Association for Molecular Pathology. Genet Med.

[10] Ellard S, Baple E, Berry I, Forrester N, Turnbull C, Owens M, Eccles D, Abbs S, Scott R, McMullan Z (2019) ACGS Best Practice Guidelines for Variant Classification 2019. Association for Clinical Genomic Science. https://www.acgs.uk.com/quality/best-practice-guidelines/

[11] Mantuano E, Romano S, Veneziano L, Gellera C, Castellotti B, Caimi S, Testa D, Estienne M, Zorzi G, Bugiani M, Rajabally YA, Barcina MJ, Servidei S, Panico A, Frontali M, Mariotti C (2010). Identification of novel and recurrent *CACNA1A* gene mutations in fifteen patients with episodic ataxia type 2. J Neurol Sci.

[12] Maksemous N, Roy B, Smith RA, Griffiths LR (2016). Next-generation sequencing identifies novel *CACNA1A* gene mutations in episodic ataxia type 2. Mol Genet Genomic Med.

[13] Reinson K, Oiglane-Shlik E, Talvik I, Vaher U, Ounapuu A, Ennok M, Teek R, Pajusalu S, Murumets U, Tomberg T, Puusepp S, Piirsoo A, Reimand T, Ounap K (2016). Biallelic *CACNA1A* mutations cause early onset epileptic encephalopathy with progressive cerebral, cerebellar, and optic nerve atrophy. Am J Med Genet A.

[14] Angelini C, Van Gils J, Bigourdan A, Jouk PS, Lacombe D, Menegon P, Moutton S, Riant F, Sole G, Tournier-Lasserve E, Trimouille A, Vincent M, Goizet C (2019). Major intra-familial phenotypic heterogeneity and incomplete penetrance due to a *CACNA1A* pathogenic variant. Eur J Med Genet.

[15] Ilg W, Bastian AJ, Boesch S, Burciu RG, Celnik P, Claassen J, Feil K, Kalla R, Miyai I, Nachbauer W, Schols L, Strupp M, Synofzik M, Teufel J, Timmann D (2014). Consensus paper: management of degenerative cerebellar disorders. Cerebellum.

[16] Gandini J, Manto M (2020). The neurological update: therapies for cerebellar ataxias in 2020. J Neurol.

[17] Strupp M, Kalla R, Dichgans M, Freilinger T, Glasauer S, Brandt T (2004). Treatment of episodic ataxia type 2 with the potassium channel blocker 4-aminopyridine. Neurology.

[18] Strupp M, Kalla R, Claassen J, Adrion C, Mansmann U, Klopstock T, Freilinger T, Neugebauer H, Spiegel R, Dichgans M, Lehmann-Horn F, Jurkat-Rott K, Brandt T, Jen JC, Jahn K (2011). A randomized trial of 4-aminopyridine in EA2 and related familial episodic ataxias. Neurology.

[19] Griggs RC, Moxley RT, Lafrance RA, McQuillen J (1978). Hereditary paroxysmal ataxia: response to acetazolamide. Neurology.

[20] Kalla R, Strupp M (2019). Aminopyridines and acetyl-dl-leucine: new therapies in cerebellar disorders. Curr Neuropharmacol.

[21] Bremova T, Malinova V, Amraoui Y, Mengel E, Reinke J, Kolnikova M, Strupp M (2015). Acetyl-dl-leucine in Niemann-Pick type C: a case series. Neurology.

